# Optimizing mesenchymal stem cell therapy: from isolation to GMP-compliant expansion for clinical application

**DOI:** 10.1186/s12860-025-00539-7

**Published:** 2025-05-06

**Authors:** Michael E. Williams, Federica Banche Niclot, Sara Rota, Jaesang Lim, Janaina Machado, Ricardo de Azevedo, Katia Castillo, Samuel Adebiyi, Ranga Sreenivasan, Daniel Kota, Patrick C. Mcculloch, Francesca Taraballi

**Affiliations:** 1 Center for Musculoskeletal Regeneration, Houston Methodist Academic Institute, Houston, TX USA; 2 Orthopedics and Sports Medicine, Houston Methodist Hospital, Houston, TX USA; 3 Ann Kimball and John W. Johnson Center for Cellular Therapeutics, Houston Methodist Academic Institute, Houston, TX USA

**Keywords:** Mesenchymal stem cells, Infrapatellar fat pad-derived MSCs, Stem cell therapy, Good manufacturing practices, Translational research

## Abstract

**Background:**

Mesenchymal stem cells (MSCs) are promising for cell-based therapies targeting a wide range of diseases. However, challenges in translating MSC-based therapies to clinical applications necessitate standardized protocols following Good Manufacturing Practices (GMP) guidelines. This study aimed at developing GMP-complained protocols for FPMSCs isolation and manipulation, necessary for translational research, by (1) optimize culture of MSCs derived from an infrapatellar fat pad (FPMSC) condition through animal-free media comparison and (2) establish feasibility of MSC isolation, manufacturing and storage under GMP-compliance (GMP-FPMSC).

**Methods:**

FPMSCs from three different patients were isolated following established protocols and the efficacy of two animal component-free media formulations in the culturing media were evaluated. The impact of different media formulations on cell proliferation, purity, and potency of MSCs was evaluated through doubling time, colony forming unit assay, and percentage of MSCs, respectively. Furthermore, the isolation and expansion of GMP-FPMSCs from four additional donors were optimized and characterized at each stage according to GMP requirements. Viability and sterility were checked using Trypan Blue and Bact/Alert, respectively, while purity and identity were confirmed using Endotoxin, Mycoplasma assays, and Flow Cytometry. The study also included stability assessments post-thaw and viability assessment to determine the shelf-life of the final GMP-FPMSC product. Statistical analyses were conducted using one-way ANOVA with Tukey’s Multiple Comparisons.

**Results:**

The study demonstrated that FPMSCs exhibited enhanced proliferation rates when cultured in MSC-Brew GMP Medium compared to standard MSC media. Cells cultured in this media showed lower doubling times across passages, indicating increased proliferation. Additionally, higher colony formation in FPMSCs cultured in MSC-Brew GMP Medium were observed, supporting enhanced potency. Data from our GMP validation, including cells from 4 different donors, showed post-thaw GMP-FPMSC maintained stem cell marker expression and all the specifications required for product release, including > 95% viability (> 70% is required) and sterility, even after extended storage (up to 180 days), demonstrating the reproducibility and potential of GMP-FPMSCs for clinical use as well as the robustness of the isolation and storage protocols.

**Conclusions:**

The study underscores the feasibility of FPMSCs for clinical uses under GMP conditions and emphasizes the importance of optimized culture protocols to improve cell proliferation and potency in MSC-based therapies.

**Supplementary Information:**

The online version contains supplementary material available at 10.1186/s12860-025-00539-7.

## Introduction

Mesenchymal stem cells (MSCs) are a key component in cell-based therapies and are increasingly recognized as active substances in cell-based medicinal products for their immunomodulatory and regenerative properties [1, 2]. Autologous and allogenic transplantation of MSCs has demonstrated significant potential for tissue regeneration in vivo. Indeed, MSCs have been isolated from donor tissue sources such as bone marrow, peripheral blood or adipose tissue, expanded in vitro, and then implanted at the site of disease of the same patient [3, 4] and their intrinsic immunomodulatory properties make MSCs an attractive candidate for tackling inflammatory conditions such as osteoarthritis [5, 6]. In allogenic implantation, MSCs have been isolated from donor tissue sources such as bone marrow, peripheral blood or adipose tissue, expanded in vitro, and then implanted at a site of disease of the same patient. However, optimal conditions for the expansion of MSCs for use in cell therapy have to be outlined to induce high levels of proliferation while maintaining stem cell characteristics including clonogenicity and the expression of specific surface markers [2].

Nowadays, the majority of MSC research has focused on cells derived from bone marrow, which are widely regarded as the gold standard [7]. However, this approach presents challenges, notably the invasive nature of bone marrow extraction, which can lead to patient morbidity [7]. This has promoted the exploration of compelling alternative MSC sources, such as infrapatellar fat pad (FP). FP-derived MSCs (FPMSCs) can be harvested with significantly less invasiveness compared to bone marrow and infrapatellar fat is often removed as surgical waste, with no additional procedures performed to specifically harvest this tissue. This ease of access may reduce patient morbidity and enhance the practicality of MSC-based therapies.

Despite the potential of MSC-therapies [8], only Ryoncil (based on Remestemcel-L) was granted approval by the Food and Drug Administration (FDA) in the USA in August 2023 for use in pediatric patients suffering from acute graft-versus-host-disease (GvHD) who are unresponsive to steroid treatment; and many are still being evaluated in clinical trials. This delay in therapy is partly due to the lack of standardized Good Manufacturing Practices (GMP) [9], coupled with the limitations of many existing protocols for the isolation and expansion of human MSCs that often rely on animal-derived supplements or enzymatic treatment. To address these challenges, animal-component-free cell media formulations have been developed, designed to meet the GMP standards and ensure the safety and efficacy of MSC therapies. These formulations are specifically designed to eliminate the inherent risks associated with animal-derived components, such as potential contamination, immunogenicity and batch-to-batch variability [10]. By providing a consistent and reliable environment for MSC expansion and culturing, these formulations not only enhance the safety of MSC but also ensure their efficacy, making them essential for the successful translation from research to clinical application.

Based on these considerations, our study aims at developing and assessing the feasibility of FPMSCs isolation and manipulation protocols and translating them from research-grade to GMP-compliant conditions, necessary step for translational research. The focus on this specific aspect of translational research—bridging preclinical studies and clinical implementation—is a necessary and underrepresented area in the field. Therefore, we firstly assess the efficacy of two animal component-free media formulations for culturing primary human FPMSCs optimizing protocols for isolation, expansion, and storage. To validate the clinical readiness of these protocols, we evaluated the reproducibility, effectiveness, and stability of FPMSC in a GMP-compliant environment (GMP-FPMSC).

While several studies have focused on MSCs derived from more traditional sources like BM or adipose tissue, the FP remains less characterized, especially regarding its feasibility for GMP-grade processing. This study represents a significant advancement in the field of MSC-based therapies, being one of the first to meticulously demonstrate the feasibility of using FPMSCs for future potential clinical trials. In addition, it serves as a foundational step for future studies and contributes actionable data to the growing body of knowledge surrounding alternative MSC sources. Indeed, by providing a robust and reproducible protocol, GMP validated, our work paves the way for the broader adoption of FPMSCs for advancing the translational potential of these cells, offering a viable and less invasive alternative to BMMSCs in regenerative medicine.

## Methods

The protocol was approved by the research ethics review committee of Houston Methodist Hospital (approval no. Pro00015718). 3 patients aged 20–24 were screened after written informed consent was obtained. Detailed inclusion and exclusion criteria applied (Table S1). Infrapatellar fat pad (IFP) tissue was acquired as waste tissue from patients undergoing anterior cruciate ligament (ACL) reconstructive surgery (Table 1) using an arthroscopic shaver and an in-line sterile collection chamber (GraftNet, Arthrex, Naples, FL, USA). Eligible patients underwent surgical excision of 10–20 g of IFP through the arthroscopic portal.

### FPMSC isolation

IFP was cut into approximately 1mm^3^ pieces prior to digestion with 0.1% collagenase in serum free media for 2 h at 37^o^C. Digested tissue was centrifuged at 300 ×g for 10 min and supernatant and surfactant removed. The cell pellet was washed with Phosphate- Buffered Saline (PBS) and filtered with a 100 μm filter. Following centrifugation at 300×g for 10 min, cell pellet was resuspended in standard MSC media containing MEM α (Thermofisher Cat# 12571063) supplemented with 10% fetal bovine serum (FBS, Atlas Cat# F-0500-A) and 20 µg/mL gentamicin (Thermofisher, Cat# 15750060). Cells were frozen at the end of 1st passage in FBS containing 10% dimethyl sulfoxide.

### FPMSC subculture

Cells from three patients (FPMSC-8, FPMSC-11, FPMSC-13) were defrosted and seeded in standard MSC media. Cells were passaged at 80-90% confluency and seeded at a density of 5 × 10^3^ cells/cm^2^. Moreover, based on their proven track records in supporting MSC expansion, maintenance and stemness under GMP-compliance conditions, the cells behavior was assessed using the following two animal component-free media formulations and compared to standard MSC media: MesenCult™-ACF Plus Medium (StemCell Technologies, Cat# 05447) and MSC-Brew GMP Medium (Miltenyi Biotec, Cat# 170-076-325). Animal component free media were prepared as per supplier’s instructions and used within 2 weeks of preparation.

### Cell doubling time

FPMSC cell doubling time for each medium was evaluated over 3 passages. Cells were seeded at a density of 5 × 10^3^ cells/cm^2^ and grown to 80 − 90% confluency. Cells were counted with a Bright-Line Hemacytometer and an inverted light microscope. The doubling time was calculated according to the following formula:

$$\:Doubling\:Time=\frac{duration*ln2}{\text{ln\:}(final\:concentration/initial\:concentration)}$$

### Colony forming units (CFU)

Colony forming capacity was assessed following FPMSC seeding at low density: 20, 50, 100 and 500 cells were seeded in 15 mm cell culture dish containing 15 ml standard or animal component-free media. Cells were grown for 10 days before staining fixing with 10% neutral buffered formalin for 30 min, washing twice with PBS and staining with 10% Crystal Violet (MilliporeSigma, Cat# V5265). Images were taken using BZ-X810 Keyence fluorescent microscope using 4X magnification objective and 12 sets of 25 images were stitched together per dish using BZ-800 analyzer software.

### MSC marker characterization

**Fig. 1:**
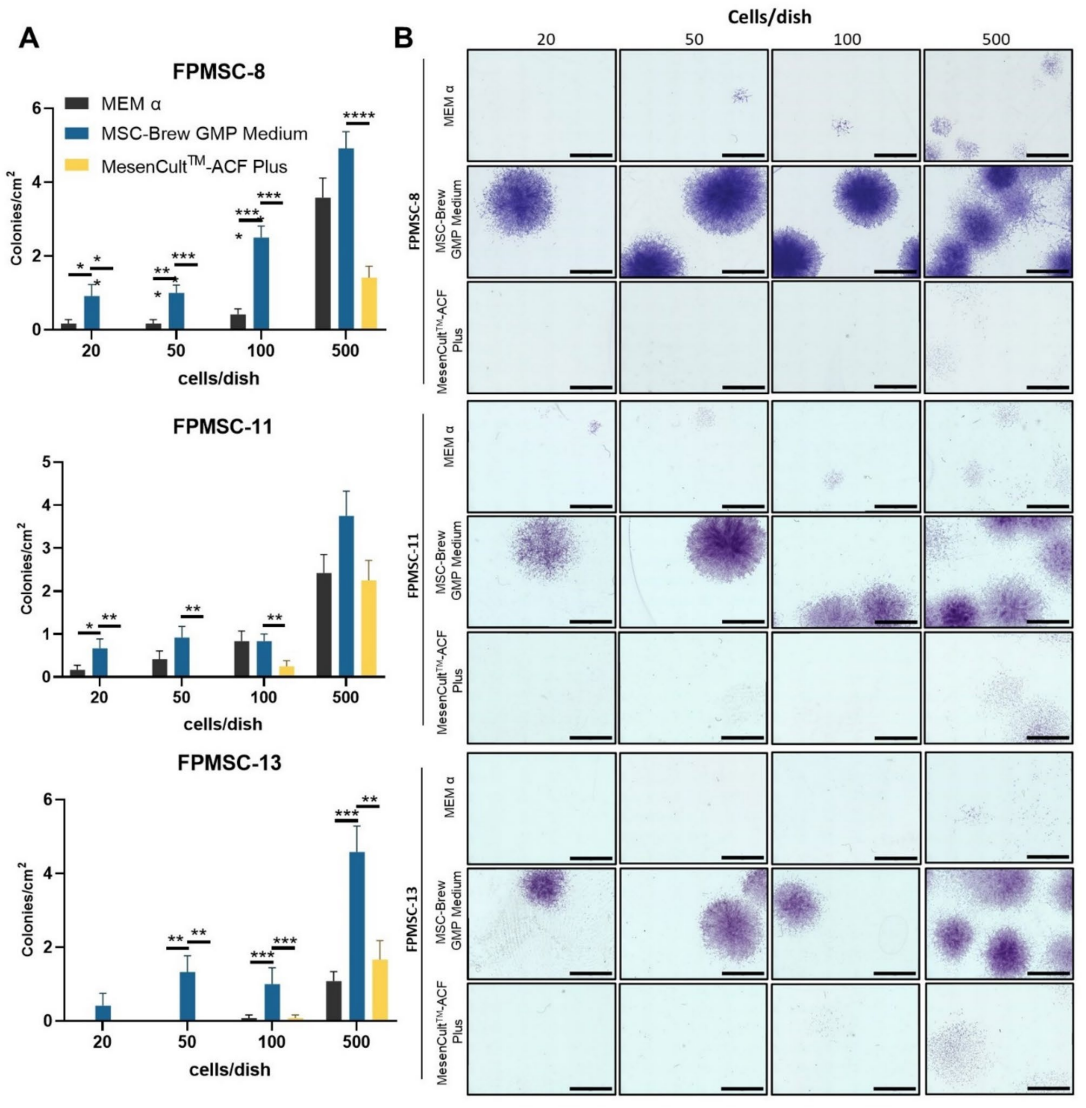
Colony forming units (CFU) were measured as number of visible colonies visible/cm^2^ (**A**) Bar plots show mean + SD, *n* = 3. *p < 0.05, **p < 0.01, ***p < 0.005, ****p < 0.0005 as indicated, one-way ANOVA with Tukey’s Multiple Comparisons. Images were comprised of 25 stitched images, representative images shown in (**B**) Scale bar = 4 mm

FPMSCs at third passage were grown for 5 days in standard MSC media or animal component-free media prior to analysis of MSC surface marker expression using BD Stemflow™ Human MSC Analysis Kit (BD Bioscienes, #562245). Flow cytometry was performed on BD FACS Fortessa using 405, 488, 561, and 630 nm lasers, gating strategy is given in Fig. 1. % MSCs, defined as CD45-, CD73+, CD90+, CD105+, was quantified using FCS Express 7 Research Edition.

### Isolation, expansion and storage of FPMSCs in GMP-compliant conditions

**Fig. 2:**
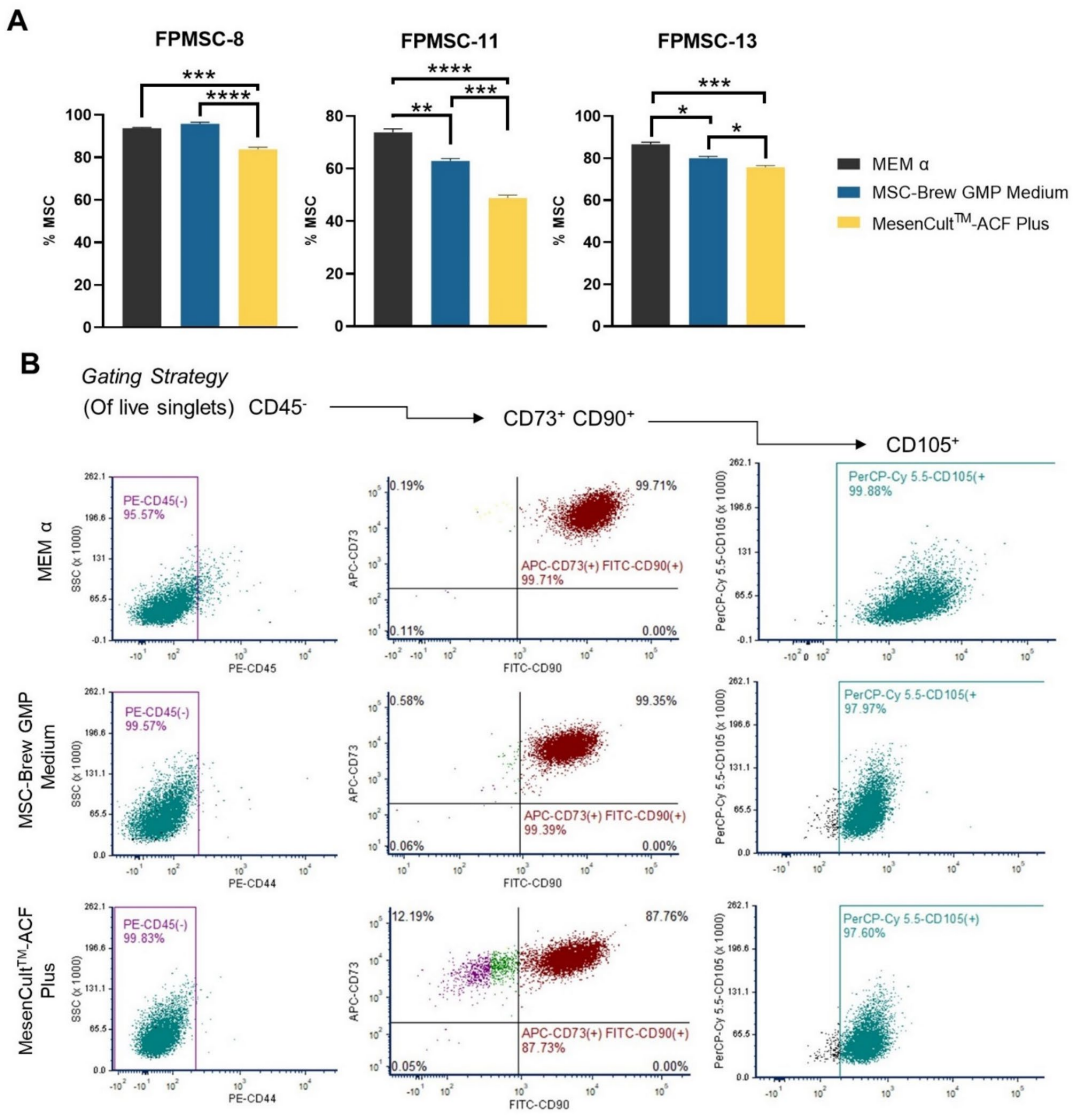
MSC marker expression in FPMSCs. MSCs were defined as CD45-, CD73+, CD90+, CD105+, determined via flow cytometry (**A**) Bar plots show mean + SD, *n* = 3. *p < 0.05, **p < 0.01, ***p < 0.005, ****p < 0.0005 as indicated, one-way ANOVA with Tukey’s Multiple Comparisons. (**B**) shows gating strategy and representative plots for FPMSC-8

Isolation of FPMSCs and their preparation for cell therapy was performed at the Ann Kimball & John W. Johnson Center for Cellular Therapy (KJCCT) which is accredited for minimal and more than minimal manipulation of cellular products by the Foundation for the Accreditation for Cellular Therapy (FACT). A flow diagram of manufacturing process and final infusion product preparation is shown in Fig. 2. The target cell dose was 5 × 10^6^ autologous FPMSCs for all donors enrolled in this study (*n* = 4).

GMP-FPMSCs were isolated and subcultured based on the procedures described above. However, additional optimization steps and rigorous controls were implemented to ensure full compliance with GMP standards.

Tissue digestion was carried out using 0.1% collagenase (Collagenase NB 6 GMP Grade) solution (Nordmark, Cat# N0002779). Cells were subcultured in MSC-Brew GMP media and detached using TrypLE™ (Gibco, Cat# 12604013) 1x solution, animal origin-free and GMP compliant alternative to trypsin. At passage 1, cells were expanded using 5-layer CellStacks − 3180 cm^2^ cell culture flask (CORNING, Cat #3311) to increase efficacy of population growth. For cryostorage, FPMSCs were frozen in cold serum- and protein-free cryopreservation solution (CS10) pre-formulated with 10% DMSO (Biolife Solutions Cat# 210202) in a 1:1 volume ratio to obtain a final concentration of 5% DMSO and 1.02 ml vial with a targeted cell count number of 5 × 10^6^ ±20% viable cells/ml. Two cryotubes of the final product are submitted to quality control tests. To maximize cell survival, an optimal cooling rate was normally achieved by using a controlled-rate freezer (Cytiva, VIA Freeze System) with a linear cooling rate of -2°C/minute until the temperature reached − 100 °C. For long term storage, cells were transferred to LN2 vapor phase storage.

### Quality control in GMP-FPMSCs

To ensure viability, safety and reproducibility in the final product, quality control (QC) measures were enacted at all stages of GMPMSC preparation (Fig. 2). Endotoxin testing was performed using the Endosafe^®^-PTS™ and a limit of ≤ 5 EU/kg/hr defined in accordance with current FDA guidelines. Sterility testing was performed using Bact/Alert 3D and MycoAlert^®^ Assay system as per manufacturer’s instructions. Cell number and viability was assessed via Trypan Blue Staining to ensure target cell dose number (5 × 10^6^ cells) and viability (> 70%) were met. The expression of HLA-DR antigen was assessed and set < 5% in order to assess the potential for safe use in clinical therapy by minimizing the risk of immune system activation. MSC immunophenotype was assessed via flow cytometry, as described above, and a threshold of > 95% set.

### GMP-FPMSC stability tests

For stability studies, GMP-FPMSC were stored at various intervals in vapor phase Liquid Nitrogen and thawed in a 37 ± 2^0^C water bath and tested for identity, purity, microbiology (mycoplasma, culture growth and gram staining), viability and count. Additionally, in order to validate the maximum interval between the thawing and administration of the product, two GMP-FPMSC tubes from two different donors were thawed and QC assessments were performed at each one hour. The final product was thawed were stored for 1, 2, 3–4 h at 2^o^-8^o^C and cultured until reaching > 70% confluence to ensure sufficient cell recovery and standardized conditions across samples.

### Statistical analysis

Differences in impact of media on doubling time, CFU and % MSCs were analyzed via one-way ANOVA with Tukey’s Multiple Comparisons using GraphPad Prism 8.

## Results

**Fig. 3:**
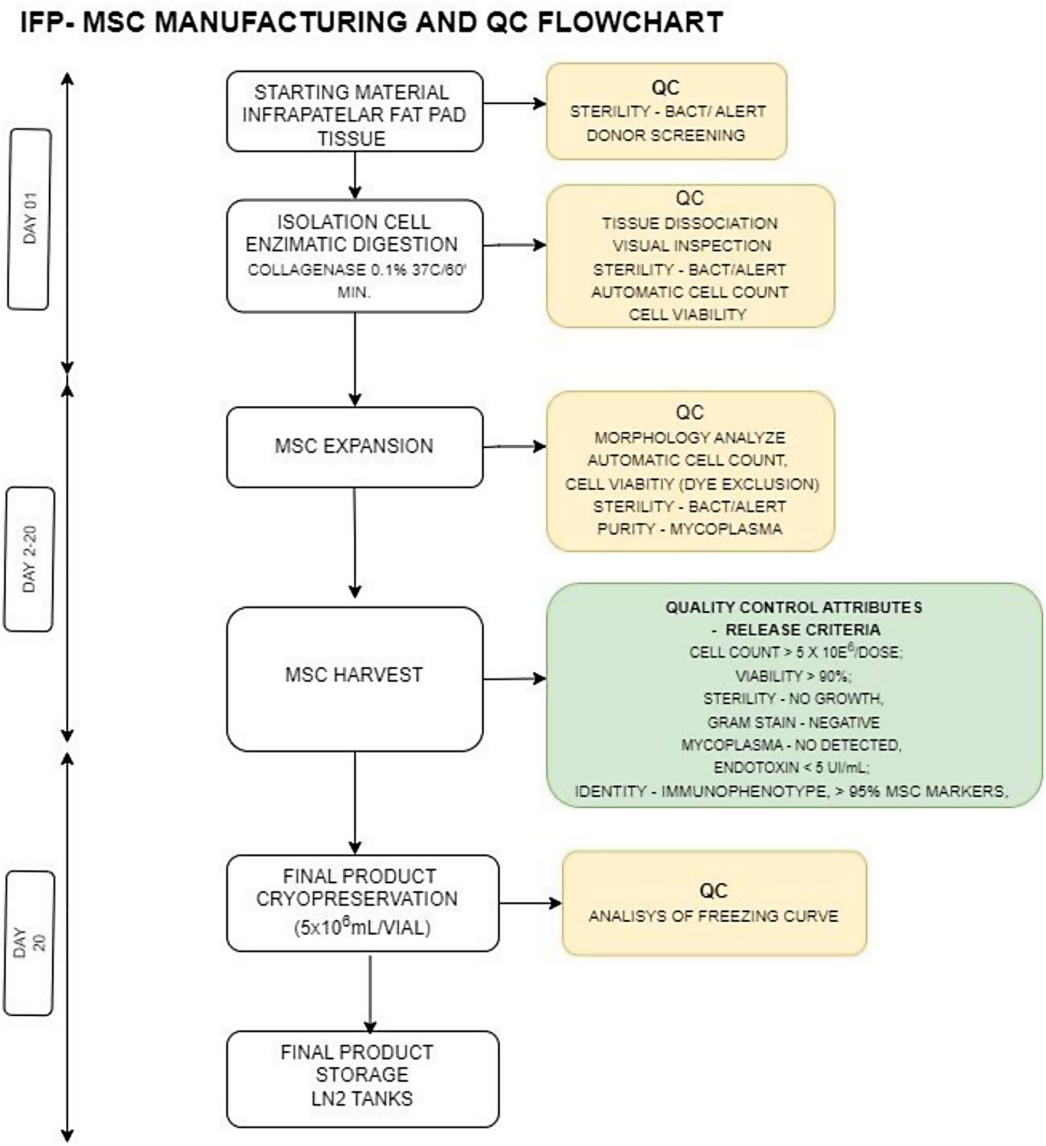
Flow diagram of FP-MSC GMP manufacturing

The capacity for primary patient MSCs to rapidly proliferate in cell culture is vital for their use in cell therapy. The doubling time for FPMSCs grown in animal component-free media was therefore assessed. The doubling time for FPMSCs derived from 3 patients (patient’ details are reported in Table 1) grown in animal component-free media formulations (supplemented MSC-Brew GMP Medium and MesenCult^TM^-ACF Plus), as well as standard MSC media (MEM α supplemented with 10% FBS), was obtained at passage 2, 3 and 4. Doubling time results are depicted in Fig. 3. FPMSCs displayed lower doubling time when grown in animal component-free media in all patients at all passage numbers and a significantly lower doubling time in MSC-Brew GMP Medium relative to MesenCult^TM^-ACF Plus during at least one passage for all patient samples, indicating a higher rate of proliferation.

**Table 1:** Donor information for FPMSC sources used in this study

Sample ID	Age	Sex	Body mass index
FPMSC-8	22	M	26.0
FPMSC-11	24	M	20.5
FPMSC-13	20	F	38.3

**Fig. 4:**
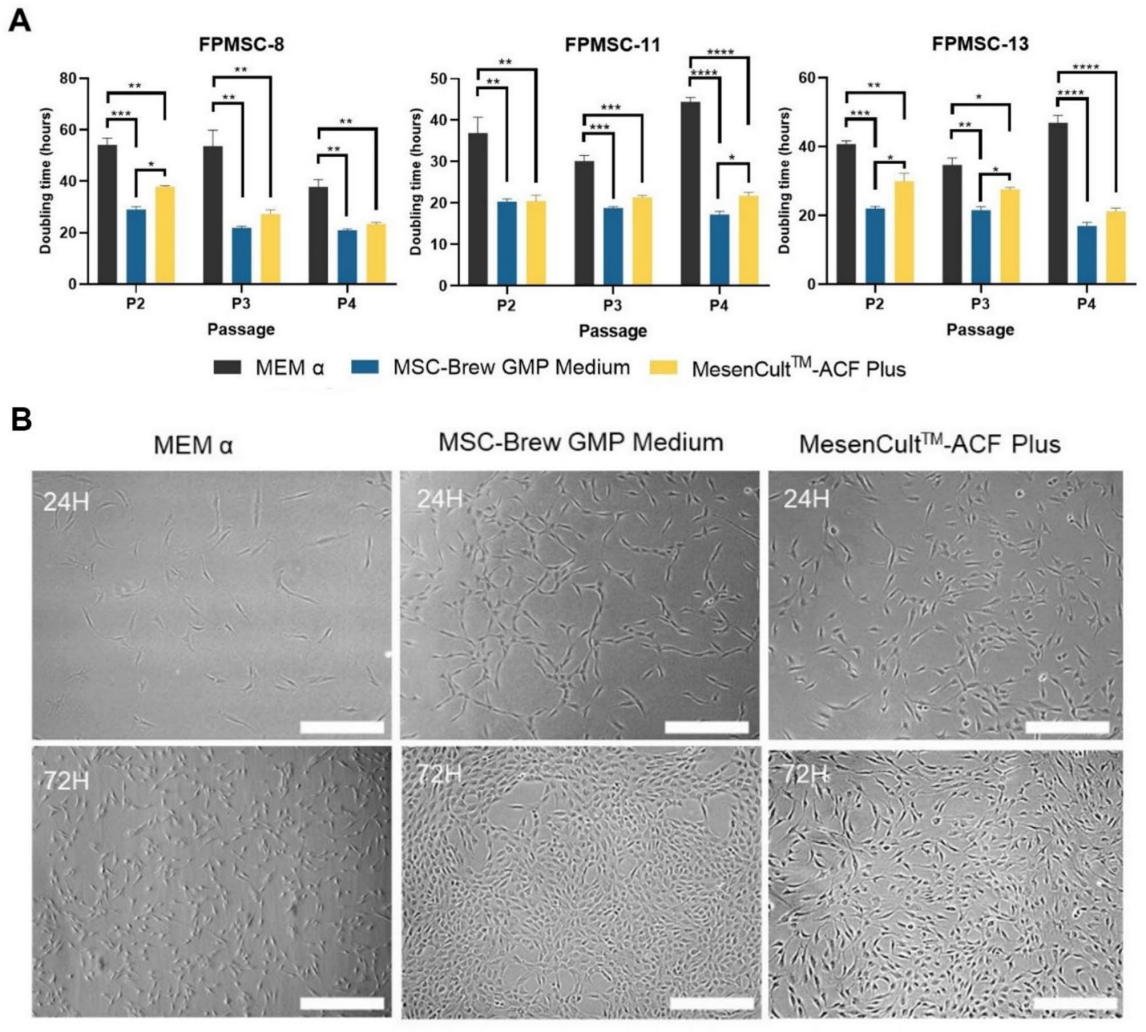
Doubling time for three patients (FPMSC-8, FPMSC-11, FPMSC-13) cultured in MEM α (supplemented with 10% FBS), MSC-Brew GMP Medium and MesenCultTM-ACF Plus at passage 2, 3 and 4 (**A**) Bar plots show mean + SD, *n* = 3. *p < 0.05, **p < 0.01, ***p < 0.005, ****p < 0.0005 as indicated, one-way ANOVA with Tukey’s Multiple Comparisons. (**B**) shows representative images of cell density for FPMSC-8 at passage 2, scale bar = 100 μm

Multipotent MSCs display the capacity for colony formation, and the potency of MSC populations can therefore be assessed via the CFU assay. Colonies formed by FPMSCs seeded at low density (20, 50, 100 and 500 cells in 15 mm cell culture dishes) and grown in standard and animal component-free media for 10 days were counted following crystal violet staining (Fig. 4A). FPMSCs grown in MSC-Brew GMP Medium consistently displayed higher colony forming capacity, while FPMSCs grown in MesenCult^TM^-ACF Plus displayed poor capacity for colony formation. Notably, colonies generated in supplemented MSC-Brew GMP Medium displayed substantially darker colonies following crystal violet staining (Fig. 4B), suggesting increased proliferation and cell density in colonies formed.

Multipotent MSCs also display a unique set of cell surface markers. To further understand the composition of MSC populations grown in animal component-free media, we analyzed MSC-specific marker expression via flow cytometry to approximate the proportion MSCs in cell populations (Fig. 1). For all patients, FPMSC populations grown in supplemented MSC-Brew GMP Medium displayed a significantly higher proportion of CD45^−^, CD73^+^, CD90^+^, CD105^+^ cell relative to MesenCult^TM^-ACF Plus.

### GMP-FPMSC feasibility

The collection of the infrapatellar tissue in the clinic, transport to KJCCT, and further FPMSC isolation, expansion, collection, and storage at KJCCT was validated under GMP conditions. GMP-FPMSCs from 4 donors (Table 2) were isolated and expanded using supplemented MSC-Brew GMP Medium as this had been shown to promote stem cell proliferation most effectively. Details of cell expansion are given in Tables 2 and 3. From a starting volume of 18 ± 3.83 ml of infrapatellar fat pad aspirate, total MSC harvest was in the range of 1–2 × 10^8^ cells after a maximum of 20 days after tissue extraction with cell viability > 95%.

**Table 2:** Outcome of FPMSC isolation and expansion under GMP requirements

Sample ID	W455623000021	W455623000032	W455623000038	W455623000040	Average
Donor age	23	42	44	50	39.7 ± 11.7
Donor sex	Male	Female	Male	Female	
Vol. starting material (ml)	20	19.5	10	24	17 ± 3.82
Initial number of cell (x10^6^)	11	2.5	5.29	3.19	5.48 ± 3.83
Initial viability (%, > 70%)	83	80	71	77	78 ± 0.05
Time until 1st passage (days)	8	14	10	10	10.5 ± 2.52
Total cells at P1 (x10^7^)	1.43	1.20	1.23	1.24	1.28 ± 0.10
Passage viability (%, > 70%)	99	89	88	96	93 ± 0.05
Total cell harvest (x10^8^)	1.55	1.05	1.00	1.01	1.15 ± 0.27
Harvest time (days)	12	20	20	16	17 ± 3.83
Cell viability at final harvest (%)	100	96	95	97	97 ± 0.02

**Table 3:** Identity of FPMSC isolation under GMP requirements

Sample ID	W455623000021	W455623000032	W455623000038	W455623000040	Average
CD73+ (%, > 95%)	97.97	97.31	98.09	98.07	97.86 ± 0.37
CD90+ (%, > 95%)	89.81	97.44	98.07	98.14	95.87 ± 4.05
CD105+ (%, > 95%)	96.77	77.17	97.97	92.93	91.21 ± 9.60
HLA-DR < 5%	0.42	0.63	1.66	1.21	0.98 ± 0.56

**Fig. 5:**
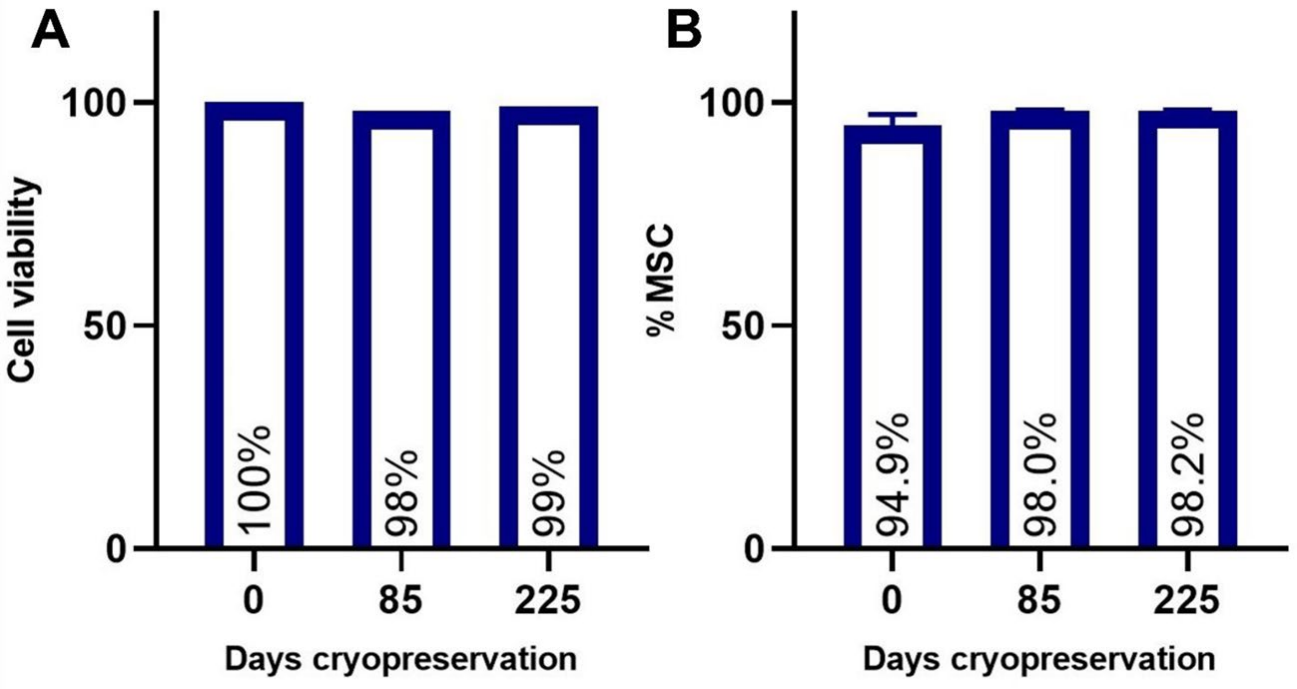
Cell viability (**A**) and stem cell marker expression (**B**) in FPMSCs following cryopreservation

Due to the nature of cell therapy, MSC populations May need to be stored for extended periods of time following isolation. Freezing and thawing of cells can have significant impact on cell viability and phenotype. Therefore, the impact of cryopreservation on GMP-FPMSC population viability and stem cell marker expression was assessed before cryopreservation (Table 3) and post-thaw (Tables 4; Fig. 5). Cells thawed and cultured following 85 and 225 days of cryopreservation did not demonstrate reduction in viability (Fig. 5A) or loss of stem cell markers (Fig. 5B)

**Table 4:** Identity and viability of FPMSC post thaw under GMP requirements

Sample ID	W455623000021	W455623000032	W455623000038	W455623000040	Average
CD73+ (%, > 95%)	99.8	99.9	99.8	99.9	99.85 ± 0.06
CD90+ (%, > 95%)	99.8	99.9	99.7	99.9	99.82 ± 0.095
CD105+ (%, > 95%)	99.8	99.7	99.5	99.9	99.72 ± 0.171
HLA-DR < 5%	2.97	1.33	0.12	0.89	1.33 ± 1.20
Viability post thaw	97.1	94.4	93.6	99.8	96.22 ± 2.81

The maximum interval between the thawing and administration of the product was also validated on two donors. As shown in Table 5, all results obtained with T0 to T 4 h samples Met the acceptance criteria. We therefore validated four hours as the maximum delay between the end of the MSCs thawing step and their administration to the patient.

**Table 5:** Viability of FPMSC between thawing and administration under GMP requirements

Sample ID	W455623000032	W455623000038
Viability T0 (> 70%)	96%	96%
Viability T1 (> 70%)	96%	95%
Viability T2 (> 70%)	96%	95%
Viability T3 (> 70%)	97%	96%
Viability T4 (> 70%)	97%	96%
Average (> 70%)	96.4 ± 0.54	95.6 ± 0.54

## Discussion

The translation of cell-based therapies from bench to bedside has proven challenging [11]. Patient-derived cell therapies will have inherently high batch-to-batch heterogeneity, meaning all other aspects of isolation and expansion of cells should be tightly controlled and highly reproducible. To date, a huge range of protocols for the isolation, expansion, storage and implantation of human MSCs have been employed using multiple tissues as cell source [8, 9]. Many media formulations for MSC expansion incorporate serum and other animal-derived components, most notably FBS, making them inadequate for use in medical products. Commercially available FBS, for example, contains many unidentified components and shows high batch-to-batch heterogeneity [12]. The use of serum-free and animal component-free formulations side-steps such limitations, as well as decreasing the risk of viral contamination from animal products. Here, we demonstrate increased proliferation and colony forming capacity of primary MSCs grown in Miltenyi’s MSC-Brew GMP Medium compared to other MSC media.

The protocol presented here represents a reliable and reproducible method through which to generate MSC populations. Furthermore, through the use of GMP-appropriate media in GMP conditions, we demonstrate its potential and readiness for use in the development of cell therapies. Each stage of isolation, storage and expansion is well characterized and optimized and can be reproduced according to GMP requirements. Furthermore, the FP is removed as waste during reconstructive knee surgery and therefore represents a reliably available donor tissue. Having demonstrated the feasibility of isolating and maintaining FPMSCs under GMP standards, future investigations will focus on confirming their retention of multipotent differentiation capabilities, as well as their safety and efficacy in a clinical setting. For example, to advance GMP-FPMSCs towards clinical application, future studies will focus on validating their genetic stability after long-term cryopreservation and thawing. This will include genomic integrity analysis across multiple manufacturing batches to ensure the safety and consistency of the final product. Moreover, future investigations will aim to extensively evaluate their multipotent capabilities, and they will foresee differentiation assays into the adipogenic, osteogenic, and chondrogenic lineages, as well as additional surface marker characterization. These assessments are essential to meet the requirements set forth by international organizations, such as FATS and International Society for Cell & Gene Therapy (ISCT), which are committed to advancing the field of cell and gene therapies. Here, we focus on the isolation and storage of FPMSCs, while future work is required to fully characterize these cells before delivery them as a therapeutic cell-based product to patients.

## Conclusions

The presented research highlighted the importance of standardized culture protocols for successful translation from laboratory settings to clinical applications, ultimately benefiting patients worldwide. We demonstrated the readiness of FPMSCs for potential use in cell-based therapies under GMP conditions and, by presenting data on viability, sterility, phenotype, and functional characteristics of FPMSCs, this manuscript highlights the feasibility of their use in clinical-grade environments. Notably, the enhanced proliferation rates and potency observed in FPMSCs cultured in animal component-free media suggested a promising avenue for improving the efficacy of MSC-based treatments. Moreover, the maintenance of viability and stem cell marker expression following cryopreservation indicates the robustness of the isolation and storage protocols, essential for long-term storage and clinical applications. Overall, this research contributes to optimizing reproducible and effective protocols for MSC isolation and expansion, emphasizing the importance of GMP compliance and standardized culture conditions in maximizing the therapeutic potential of MSCs in regenerative medicine.

## Electronic supplementary material

Below is the link to the electronic supplementary material.

Supplementary Material 1

## Data Availability

No datasets were generated or analysed during the current study.

## Abbreviations

ACL: Anterior cruciate ligament
KJCCT: Ann Kimball & John W. Johnson center for cellular therapy
CFU: Colony forming units
FPMSC: Fat Pad-derived MSCs
FBS: Fetal bovine serum
FDA: Food and drug administration
FACT: Foundation for the accreditation for cellular therapy
GMP-FPMSC: FPMSC manipulated under GMP-compliance
GvHD: Graft-versus-host disease
GMP: Good manufacturing practices
IFP: Infrapatellar fat pad
ISCT: International society for cell & gene therapy
MSC: Mesenchymal stem cells
PBS: Phosphate-buffered saline
QC: Quality control
