# Supplementary material for: Optimizing mesenchymal stem cell therapy: from isolation to GMP-compliant expansion for clinical application
Source: BMC Mol Cell Biol. 2025 May 6;26:15. doi: 10.1186/s12860-025-00539-7 (PMC12054297; doi:10.1186/s12860-025-00539-7)
Supplement: Supplementary file 1 — Supplementary Material 1 [file 12860_2025_539_MOESM1_ESM.docx]

**Optimizing Mesenchymal Stem Cell Therapy: From Isolation to GMP-Compliant Expansion for Clinical Application**

*Michael E. Williams^1,2,^, Federica Banche-Niclot^1,2^, Sara Rota^1,2^, Jacob Lim^1,2^, Janaina Machado^3^, Ricardo de Azevedo^3^, Katia Castillo^3^, Samuel Adebiyi^3^, Ranga Sreenivasan^3^, Daniel Kota^3^, Patrick C. Mcculloch^2^ and Francesca Taraballi^1,2^*.*

*^1^Center for Musculoskeletal Regeneration, Houston Methodist Academic Institute, Houston, TX, USA*

*^2^Orthopedics and Sports Medicine, Houston Methodist Hospital, Houston, TX, USA*

*^3^Ann Kimball & John W. Johnson Center for Cellular Therapeutics, Houston Methodist Academic Institute, Houston, TX, USA*

*Corresponding author: ftaraballi2@houstonmethodist.org*

*Supplementary Information*

| Eligibility Criteria |
| --- |
| Description |
| Inclusion Criteria: |
| 1. Male or Female, aged 18-70 years |
| 1. Undergoing ACL reconstruction and/or knee arthroscopy |
| 1. Full understanding and willingness to comply with fat harvesting, laboratory tests, and diagnostic imaging |
| All inclusion criteria will be determined by patient history and/or by the blood test taken during surgery. If a test for the conditions previously listed comes back positive, the patient will be notified, and the sample will be properly disposed of. |
| Exclusion Criteria: |
| 1. Pregnancy, nursing or plans for pregnancy (verified by preoperative pregnancy testing as part of standard care) |
| 1. Body Mass Index (BMI)>35 |
| 1. Significant congenital or acquired knee deformity |
| 1. Presence of orthopedic hardware or implants in the target knee |
| 1. Use of chronic immunosuppressive therapy or systemic steroids, or conditions causing immunosuppression |
| 1. Active infections in either lower extremity |
| 1. Systemic inflammatory or rheumatological conditions (e.g., rheumatoid arthritis, systemic sclerosis, lupus, or Ehlers-Danlos Syndrome) or infectious diseases (e.g., tuberculosis, HIV, hepatitis, syphilis) |
| 1. Cancer/malignancy history, except adequately treated basal or squamous cell carcinoma not involving the knee |
| 1. Contraindication to MRI or unwillingness to undergo MRI procedures |
| 1. Current use of tobacco or nicotine substitutes |
| 1. History or current evidence of substance or alcohol abuse, recreational drug use, misuse of prescription drugs, or use of medical marijuana within 30 days |
| 1. Any condition deemed by the investigator to interfere with compliance, safety, or the interpretation of study results |

***Table S1.*** *Eligibility criteria applied in this study.*
